# Adsorption of Cadmium on Degraded Soils Amended with Maize-Stalk-Derived Biochar

**DOI:** 10.3390/ijerph15112331

**Published:** 2018-10-23

**Authors:** Caixia Wu, Yungui Li, Mengjun Chen, Xiang Luo, Yuwei Chen, Nelson Belzile, Sheng Huang

**Affiliations:** 1 Department of Environmental Engineering, School of Environment and Resource, Southwest University of Science and Technology, Mianyang 621010, China; caixwugreen@163.com (C.W.); kyling@swust.edu.cn (M.C.); lx_luoxiang@163.com (X.L.); ychen@laurentian.ca (Y.C.); nbelzile@laurentian.ca (N.B.); hs1973@126.com (S.H.); 2 Low-cost Wastewater Treatment Technology International Sci-Tech Cooperation Base of Sichuan Province, Mianyang 621010, China; 3 Key Laboratory of Solid Waste Treatment and Resource Recycle, Ministry of Education, Southwest University of Science and Technology, Mianyang 621010, China; 4 Department of Chemistry and Biochemistry, Laurentian University, Sudbury, ON P3E 2C6, Canada

**Keywords:** soil, degradation, biochar, cadmium, adsorption

## Abstract

Biochar has been extensively proven to distinctively enhance the sorption capacity of both heavy metal and organic pollutants and reduce the related environmental risks. Soil pollution and degradation widely coexist, and the effect of biochar addition on adsorption behavior by degraded soils is not well understood. Four degraded soils with different degrees of degradation were amended with maize-stalk-derived biochar to investigate the adsorption of cadmium using batch methods. The maximum adsorption capacity (*Q*_m_) of degraded soil remarkably decreased in comparison with undegraded soil (5361 mg·kg^−1^→170 mg·kg^−1^), and the *Q*_m_ of biochar increased with increasing pyrolysis temperature (22987 mg·kg^−1^→49016 mg·kg^−1^) which was much higher than that of soil. The addition of biochar can effectively improve the cadmium adsorption capacity of degraded soil (36–328%). The improving effect is stronger when increasing either the degradation level or the amount of added biochar, or the pyrolysis temperature of biochar. Contrary to the general soil–biochar system, adsorption of Cd was not enhanced but slightly suppressed (7.1–36.6%) when biochar was incorporated with degraded soils, and the adsorptivity attenuation degree was found to be negatively linear with SOM content in the degraded soil–biochar system. The results of the present study suggest that more attention on the adsorption inhibition and acceleration effect difference between the soil–biochar system and the degraded soil–biochar system is needed.

## 1. Introduction

Soil pollution has received more and more attention in the past few decades [1], as it not only makes fertility of the soil drop, but also makes the yield and quality of crops decline [2,3]. Cadmium (Cd) is a metal with strong biological toxicity [4,5,6], high mobility, and long-lasting residence time. Cd is the most prominent metal contaminant in the soil in China; 7.0% of surveyed soil has surpassed the national threshold for Cd [7]. In recent years, the amounts and usages of Cd have been greatly increasing [8]. It is generally believed that sewage irrigation, application of the mineral fertilizers, toxic metals discharge from industries, and emissions from automobile exhaust are the main causes of Cd-polluted soil [9]. Adsorption behavior of pollutants in soil influences the bioavailability and environmental risk of pollutants. The ecological risks and mobility of Cd for crops reduces if Cd is adsorbed [10].

Coexisting with soil pollution, soil degradation is also a serious problem. Soil degradation is a result of natural process and human intervention. By the end of 2014, the total area of soil erosion, land desertification, and land sandification in China was 2,949,000 km^2^, 2,611,600 km^2^, and 1,721,200 km^2^, respectively [11]. As soil degrades, the nature of soil is remarkably changed, including destroyed agglomerating structure and particles, lower organic matter content, and poorer microbial communities, which makes the water content and environmental buffer capacity drop [12,13]. The natural change of degraded soil also influences the adsorption behavior of pollutants. However, the effect of degradation on adsorption of soil is not well understood. Theoretically, pollutant adsorption capacity is also reduced due to the reducing of soil organic matter content [14,15]. The mobility and bioavailability of pollutants in the soil solution increase, and consequently the environmental and ecological risks rise [16].

Biochar is a common environmentally friendly amendment material for enhancing soil adsorption performance and has good application prospects [17,18,19]. Biochar is a carbon-rich solid produced by low-temperature pyrolysis (<700 °C) of biological waste under anoxic or anaerobic conditions. Biochar possesses a good porous structure, a large specific surface area (<210 m^2^·g^−1^), and various surface oxygen-containing functional groups [18,20]. These excellent physical and chemical properties contribute to the adsorption and immobilization of pollutants in soil [21]. Adding a small amount (≦5%) of biochar (vinegar residue biochar; Maize stover biochar) to contaminated soil can effectively reduce the concentration of pollutants in the porewater of soil and decrease the migration of pollutants and its accumulation in plants and animals [21,22,23,24]. Bian et al. [18] showed that the Cd content in rice from soils supplemented with wheat straw biochar was reduced by 20% to 90%, and the Cd content of rice was less than 0.4 mg·kg^−1^. Li et al. [21] found that bioavailable Cd decreased most with 5% vinegar residue biochar application in soil. Liu et al. [25] found that, when adding 5% (in mass) stalk carbon with particle size of 0.25 mm in paddy soil, available contents of Pb, Zn, and Cu in the soil were reduced by 52.5%, 52.1%, and 50.1%, respectively. Adsorption of heavy metals (such as Cd, Pd, and Cr) usually increased in soil–biochar system due to the increasing sorbed sites for heavy metals which was supplied by the coated DOM (dissolved organic matter) of soil onto biochar [26,27]. In theory, biochar addition can improve the adsorption capacity of degraded soil and reduce the mobility and bioavailability of pollutants.

In this study, four degraded soils with different degrees of degradation were selected to investigate the effect of degradation on adsorption of cadmium while Peat and nondegraded soil were chosen as the controls. Four degraded soils were amended with maize-stalk-derived biochar to illustrate the effect of biochar addition on adsorption of cadmium onto degraded soil.

## 2. Materials and Methods

### 2.1. Soil and Biochar Samples Preparation

The subalpine meadow soil samples were collected from Hongyuan County, Aba Tibetan, and Qiang Autonomous Prefecture of the Sichuan Province (the detailed location information is shown in Table 1). According to GB19377-2003 (Chinese standard), the degraded soils were divided into different degraded levels (LDSI: slightly degraded soil I, LDSII: slightly degraded soil II, MDS: moderately degraded soil, SDS: severely degraded soil). As the controls of degraded soils, the Peat and nondegraded soil (NDS) were also collected from Hongyuan County. Random sampling was performed and two quadrats for each location were chosen. A quadrat size was 10 m × 10 m and three sampling points were randomly selected within each quadrat. The sampling depth was from 0 to 10 cm. After thoroughly homogenized, the three soil samples were stored in a soil sample box and were taken back to the laboratory. After air-drying, the soil was milled and sieved through a 100 mm screen, gravel, dead leaves, and other impurities were removed, and the samples were sealed and stored.

The fresh maize stalk was first crushed with an impact mill then placed in an oven at 100 °C for 24 h (MS100). Using an oxygen-limited pyrolysis method, the compacted maize stalk powders of grams were placed in a crucible, covered with a lid, and put into a muffle. The final carbonization temperature gradient was set at 350 °C, 500 °C, and 700 °C, respectively, at the heating rate of 5 °C·min^−1^ and for a total time of 6 h. The samples were then cooled to room temperature and the obtained biochar samples with different pyrolysis temperatures were placed in a drying tower. The biochar samples were named as MS350, MS500, and MS700, respectively.

### 2.2. The Characterization of Soil and Biochar

The soil moisture content was measured by the drying method (drying to constant weight at 105 °C) and the content of soil organic carbon (TOC) was measured by Total Organic Carbon Analyzer (Elementar Analysensyteme GmbH, Langenselbold, Germany) after carbonate removal in soil samples.

The yield and ash content of the biochar were determined according to the method GB/T17664-1999 and GB/T12496.3-1999 (Chinese standard). The CHN percentage of the biochar was determined by using a CHN elemental analyzer (Elementar Analysensyteme GmbH, Langenselbold, Germany). Each sample was measured in duplicate and the average value was given. The content of O element was calculated by subtraction.

The pH was measured by a pH meter (PHS-2C Precision Acidity Meter, Shanghai Jingke Leici, Shanghai, China) in a solid–liquid ratio of 1:2.5 with distilled water. The specific surface area (SSA) of the soil (before and after Cd adsorption) was determined by the N_2_ adsorption method according to the BET theory (ST-08 specific surface area analyzer, Quantachrome Instruments, Boynton Beach, FL, USA). The infrared spectrum (before and after Cd adsorption) was determined by FTIR (PerkinElmer Instrument Co., Ltd., Waltham, MA,. USA), and the surface topography (before and after Cd adsorption) was determined by SEM (Zeiss AG, Oberkochen, Germany).

### 2.3. Isothermal Adsorption Experiment

The isothermal adsorption curve of Cd on each sample was obtained by bulk adsorption experiments. The adsorption experiments were carried out under the following conditions: pH = 7.0, 0.01 mol·L^−1^ CaCl_2_ and 200 mg·L^−1^ NaN_3_ as background solution, of which the CaCl_2_ was for the ionic strength control and NaN_3_ was to inhibit the microorganisms and prevent the related adsorption or absorption of Cd. The isothermal adsorption experiments were performed by accurately weighing (BSA224S Electronic Balance, Beijing Sartorius Scientific Instrument Company, Beijing, China) a sample amount and adding 8.0 or 20.0 mL of Cd solution at different concentrations (0, 2.5, 5, 7.5, 10, 15, 20, 40, 80, 120, 160 mg L^−1^). At each concentration, two blank controls (without added samples) were run for each sample. After mixing the sample with the Cd solution, the vial was immediately covered with a PTFE gasket lid. The solution was shaken at 150 r·min^−1^ for 24 h (ZWY-211C thermostatic oscillator, Shanghai Zhicheng Analysis Instrument Co., Ltd., Shanghai, China) without light at a constant temperature of 10 ± 1 °C. After the adsorption was completed, the supernatant was withdrawn from each vial after centrifugation at 4000 r·min^−1^ for 20 min (TGL-16C Desktop High Speed Centrifuge, Shanghai Anting Scientific Instrument Factory, Shanghai, China). Then, the Cd content in the solution was measured by flame atomic adsorption spectrometry (PE900T Flame Atomic Adsorption spectrometer, PerkinElmer Instruments) after dilution with 1% HNO_3_. The detection wavelength was at 228.8 nm and the detection limit was 0.005 mg·L^−1^.

An appropriate solid–liquid ratio was selected based on the results of the pretests. For the isothermal adsorption experiments of Cd on different degradations of soil alone, the mass of samples was between 300 and 1500 mg of soil, to which 20.0 mL of Cd solution was added. For the experiments with biochar alone, 8–40 mg of biochar was used with a presence of 8.0 mL of Cd solution. For experiments with a combined biochar and soil system, three sets of tests were conducted: (1) with 500 mg moderately degraded soil (MDS) in presence of 20.0 mL of Cd solution at different initial concentrations and 1.0%, 2.0%, and 4.0% (wt) of biochar MS700, respectively; (2) with 500 mg of MDS in 20.0 mL of Cd solution and 2.0% (wt) of MS100, MS350, MS500, and MS700, respectively, and (3) with 200–500 mg of different levels of degraded soil in presence of 2.0% (wt) MS700 and 20.0 mL of Cd solution.

All chemicals were of analytical grade, including Cd(NO_3_)_2_·4H_2_O, CaCl_2_ and NaN_3_, and HNO_3_. The experimental water was ultrapure water (Millipore, Danvers, MA, USA).

### 2.4. Data Processing

The amount of Cd adsorbed (*Q*_e_) by adsorbent (soil, biochar, or both) at equilibrium can be calculated as follows:

(1) $$Q_{e}=\frac{1000(C_{o}-C_{e})V}{m}\;$$

where *Q*_e_ (mg·kg^−1^) is the adsorption amount at equilibrium; *C*_o_ (mg·L^−1^) and *C*_e_ (mg·L^−1^) are the initial and equilibrium concentrations in the solution after adsorption; *V* (mL) is the volume of the solution in the experiment, and m (mg) is the mass of the experimental sample.

The Freundlich Equation (2) and Langmuir Equation (3) isothermal models were used to fit the adsorption data.

(2) $$Q_{e}=K_{f}{C_{e}}^{N}\;$$

(3) $$Q_{e}=\frac{Q_{m}K_{L}C_{e}}{1+K_{L}C_{e}}\;$$

where *K*_f_ ((mg·kg^−1^)·(mg·L)^−N^) is the Freundlich regression parameter; *N* is the Freundlich adsorption constant; *Q*_m_ (mg·kg^−1^) is the maximum adsorption capacity; *K*_L_ (L·mg^−1^) is a constant related to the binding strength; *Q_e_* and *C_e_* are the same definitions as in Equation (1).

For a mixed adsorption system, the adsorption contribution of Cd by the biochar and soil is assumed to be a simple summation (*Q’*_m,soil+BC_), and the sum of adsorption capacity can be expressed by the simple mathematical addition as given in the formula Equation (4).

(4) $$Q_{m,\mathrm{soil}+\mathrm{BC}}^{\prime }=Q_{m,\mathrm{soil}}\times f_{\mathrm{soil}}+Q_{m,\mathrm{BC}}\times f_{\mathrm{BC}}\;$$

where *Q’*_m,soil+BC_ (mg·kg^−1^) represents the total adsorption amount in a mixed system calculated based on the assumption; *Q*_m,soil_ (mg·kg^−1^) and *Q*_m,BC_ (mg·kg^−1^) are the adsorption amounts calculated according to Equation (3), and *f*_soil_ (%) and *f*_BC_ (%) are the weight fraction of soil and biochar, respectively (*f*_soil_ + *f*_BC_ = 1).

Due to the low proportion of added biochar in a soil sample and the complex composition of soil, the adsorption efficiency of the biochar after being mixed into the soil was lower than that in a pure biochar system, and the degree of decline can be expressed by Equation (5):

(5) $$D\%=\frac{Q_{m,\mathrm{soil}+\mathrm{BC}}^{\prime }-Q_{m,\mathrm{soil}+\mathrm{BC}}}{Q_{m,\mathrm{soil}+\mathrm{BC}}^{\prime }}\times 100\;$$

where *D* (%) is the relative adsorption decline level of Cd adsorption in the soil amended with biochar; *Q*’_m,soil+BC_ (mg·kg^−1^) is the calculated Cd adsorption capacity in soil and biochar system at equilibrium according to Equation (4), and *Q*_m,soil+BC_ (mg·kg^−1^) is the maximum adsorption amount obtained from experimental isotherm of a soil and biochar mixed system.

(6) $$RC_{i}\%=\frac{f_{i}Q_{m,i}}{Q_{m,\mathrm{soil}+\mathrm{BC}}}\times 100\;$$

where *RC*_i_ (%)is the adsorption relative contribution of soil and biochar (*i* = 1, 2. 1 represents soil; 2 represents biochar); *f*_i_ (%) is the weight fraction of soil and biochar, respectively, and *Q*_m,i_ (mg·kg^−1^) is the adsorption amount calculated according to Equation (3); *Q*_m,soil+BC_ (mg·kg^−1^) represents the total adsorption amount in a mixed system calculated based on the assumption.

## 3. The Results and the Discussion

### 3.1. The Characterization of the Tested Pristine Soils and Biochars

**Figure 1:**
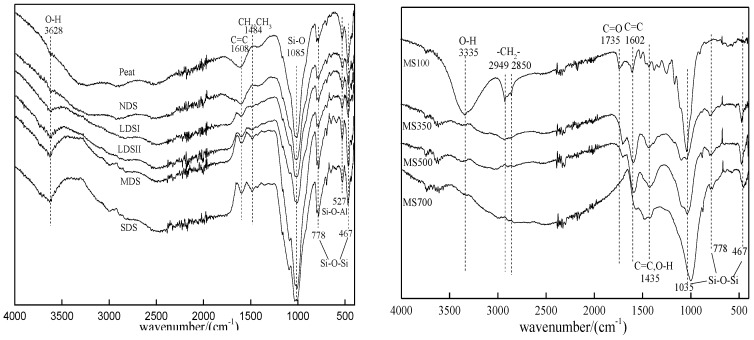
The FTIR spectra of the six selected soils and biochar.

**Figure 2:**
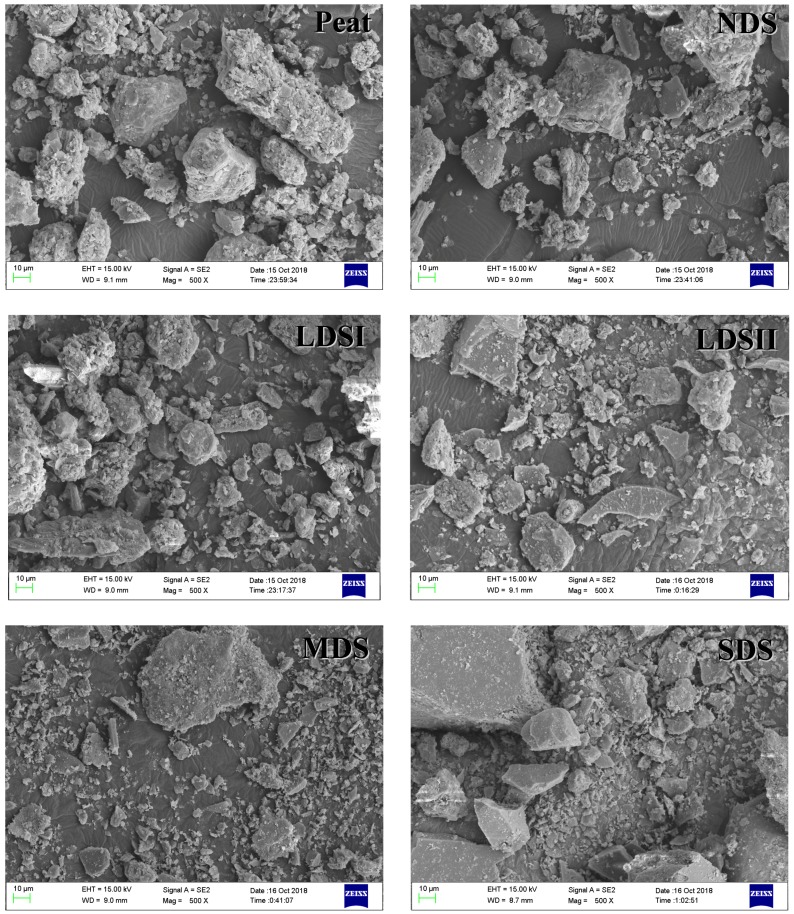
The SEM images of the six selected soils.

The basic physicochemical properties of the six tested pristine soils are shown in Table 1. With the soil degradation progress, the soil moisture content decreased by 18.7 times, and pH value increased from 5.72 to 6.91. The specific surface area (SSA) was relatively close (2.02–3.11 m^2^·g^−1^). The total organic carbon (TOC) content was sharply reduced with the degradation of soil from 92.7 g·kg^−1^ in Peat to 5.60 g·kg^−1^ in SDS, for a reduction of nearly 16 times [28]. The sharp decline of TOC in the study area was the result of the lack of replenished organic carbon and the continuously increasing organic carbon mineralization with the soil degradation process. Under the overfeeding of livestock in the local area, the growth and development of herbaceous plants were inhibited. The vegetation litter was reduced and the soil organic matter could not be replenished. At the same time, the local wind erosion and freeze–thaw continuously accelerated the process of mineralization of soil organic matter [29,30]. The FTIR spectrum and SEM image of six tested soils are shown in Figure 1 and Figure 2. The FTIR spectrum showed peaks at 3628 cm^−1^, 1484 cm^−1^, 778 cm^−1^, 527 cm^−1^, and 467 cm^−1^ due to presence of O–H, CH_2_, CH_3_ Si–O–Si, Si–O–Al, and Si–O–Si groups, respectively [31], and the vibration intensity decreased with the soil degradation process. A strong band inversion of the Si–O stretching was evident from the peak at 1085 cm^−1^ of MDS. The SEM images showed amounts of soil organic matter and large aggregates in Peat and NDS. With the soil degradation process, the microaggregate and mineral ratio increased in LDSI, LDSII, MDS, and SDS.

**Figure 3:**
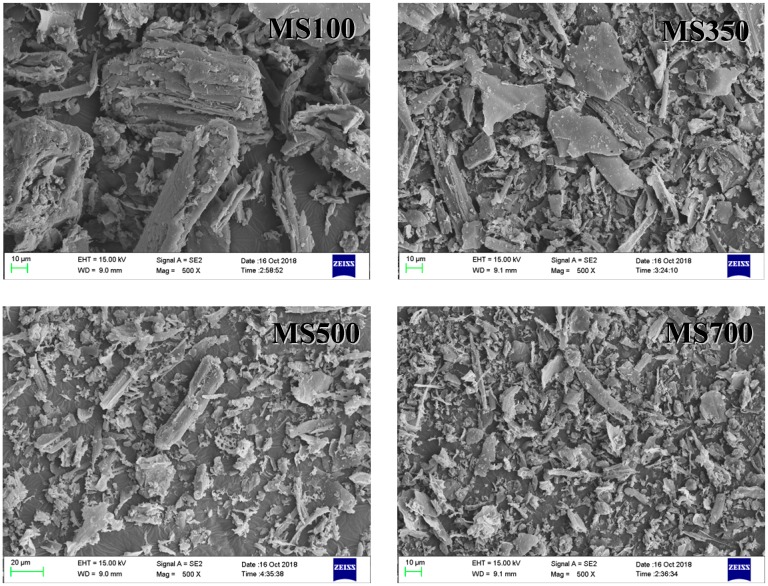
The SEM images of biochar.

The main physicochemical properties of the biochar are shown in Table 2. When the pyrolysis temperature of biochar rose from 350 °C to 700 °C, the ash content slightly increased and the specific surface area (SSA) increased by 33.9 times. The MS100 particle size (d_50_) was 11.36 μm. With the increase of the pyrolysis temperature, the d_50_ particle size was obviously reduced. It means that when temperature increases, the pyrolysis of cellulose, hemicellulose, and lignin in the biomass material is more complete. As the pyrolysis temperature increased from 350 °C to 700 °C, the contents of H, N, and O all decreased, while that of C increased by 13.3%. The aromaticity and polarity of the biochar adsorbent could be estimated through the atomic ratio H/C and (O+N)/C. The aromaticity is higher with smaller H/C ratio, and the greater polarity is with higher (O+N)/C ratio. As the pyrolysis temperature increased, the aromaticity of biochar increased (H/C, 1.7–0.36) and the polarity decreased [(O+N)/C, 0.82–0.26] [33,34]. The FTIR spectrum and SEM of the biochar are shown in Figure 1 and Figure 3. The FTIR spectrum of MS100 showed peaks at 3335 cm^−1^, 2949 cm^−1^, 2850 cm^−1^, and 1735 cm^−1^ due to presence of O–H, CH_2_, CH_2_, and C=O groups, respectively [35], and the peak disappeared with the increasing of the pyrolysis temperature. However, the aromatic group C = C (1602 cm^−1^) was retained. The peaks at 1035 cm^−1^, 778 cm^−1^, and 467 cm^−1^ may be attributed to Si–O–Si vibration, and the vibration intensity increased as the pyrolysis temperature increased. Comparing with biochar, the biomass (MS100) showed loosening and a lamellar structure with large particles. With the increase of carbonization temperature, the bulk structure was broken and the ratio of granule increased in the biochar, which was consistent with the results of the BET characterization.

### 3.2. The Cd Adsorption Isotherms on Degraded Soils

**Figure 4:**
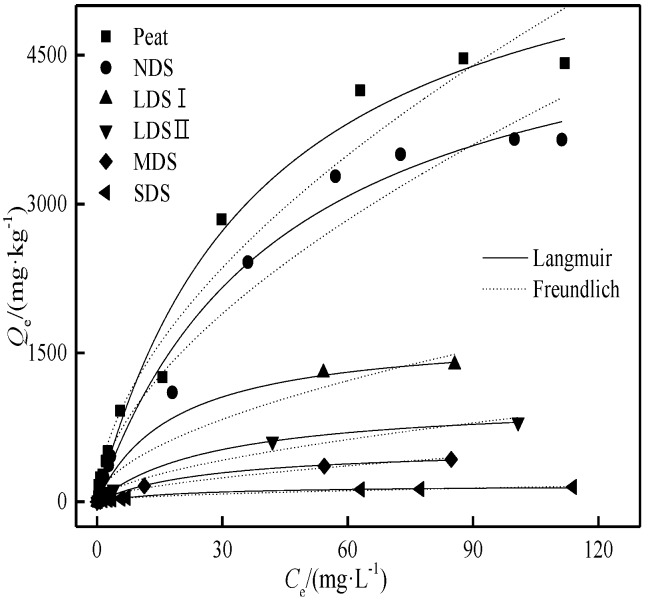
The Cd adsorption isotherms on the six selected soils.

The isothermal adsorption curves of Cd in the four degraded soils, Peat, and NDS are shown in Figure 4, and the regression parameters obtained from the Langmuir and Freundlich models are shown in Table 3. The adsorption data can fit well with both the Langmuir and Freundlich equations (*R*^2^ ≥ 0.97). The Freundlich parameter *N* value varies between 0.500 and 0.584. It is known that the nonlinearity is obvious. From the isotherm adsorption curve, it is observed that the adsorption amount decreased with the soil degradation.

According to Table 3, the Langmuir maximum adsorption capacity (*Q*_m_) of Peat and NDS were 6309 mg·kg^−1^ and 5361 mg·kg^−1^, respectively. As expected, the Langmuir maximum adsorption capacities of Cd decreased remarkably in the following order: LDSI (1710 mg·kg^−1^) > LDSII (1028 mg·kg^−1^) > MDS (553 mg·kg^−1^) > SDS (170 mg·kg^−1^). Adsorption capacity of SDS declined 29.5 times compared with NDS, which made the mobility and bioavailability of Cd in the SDS solution increase. The Cd adsorption capacity of soils is found to range from 545 to 2675 mg·kg^−1^ [36,37,38]. The adsorption capacities of degraded soils in the current research were comparable to that of the other soils, and NDS and Peat were higher than that of the other soils.

**Figure 5:**
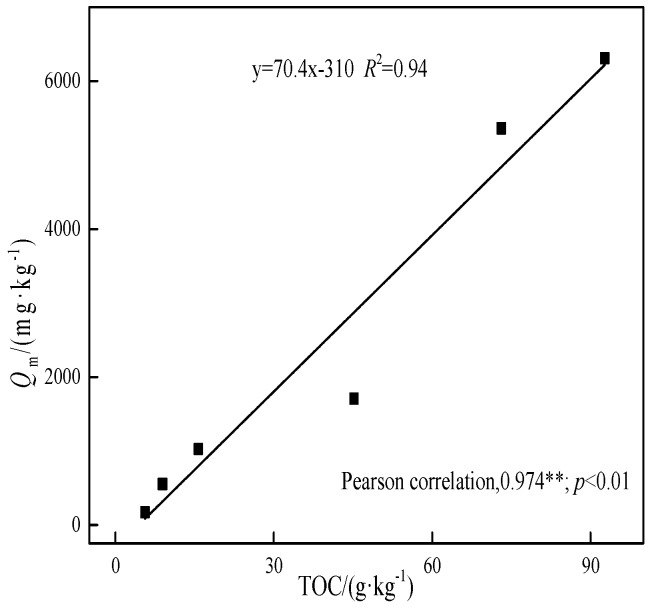
The relationship between the maximum adsorption capacity of cadmium on the six selected soils and TOC in the soils.

The huge difference in adsorption behavior of the five soil samples and Peat is hard to attribute to the specific surface area (2.02–3.11 m^2^·g^−1^). As imagined, the sharp decrease of the adsorption capacity of degraded soils is closely related to the TOC of the soils, as shown in Figure 5, where an excellent coefficient of correlation between *Q*_m_ and TOC is found (*R*^2^ = 0.94). It demonstrates the important role of soil organic material in retention and immobilization of Cd in soil [14,15].

### 3.3. The Cd Adsorption Isotherms with the Biochars

**Figure 6:**
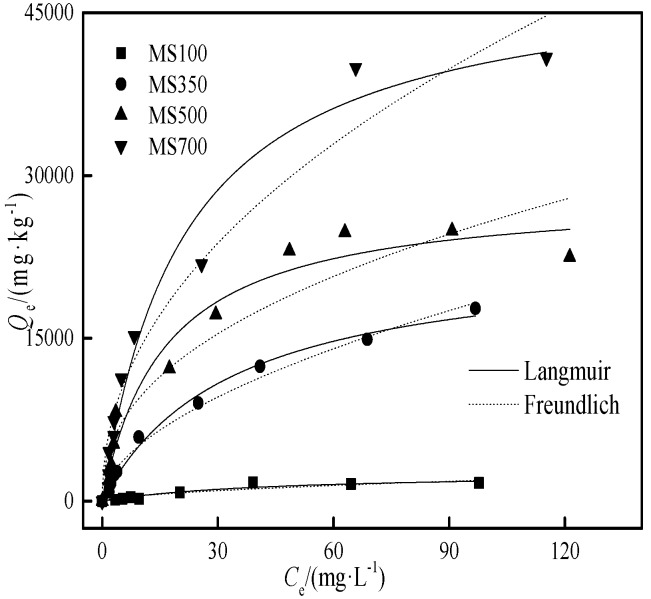
The Cd adsorption isotherms of the biochars.

The Cd adsorption isotherms on the maize stalk biochar is shown in Figure 6, and the fitting parameters of the Langmuir and Freundlich models are given in Table 3. The results show that Cd adsorption data is fit better to the Langmuir model on biochar (*R*^2^ ≥ 0.92). At the same initial concentration, the adsorption of Cd and its adsorption capacity were increased with the pyrolysis temperature of biochar.

As the control of biochar, the Langmuir maximum adsorption amount of MS100 is 2622 mg·kg^−1^. With the increase of carbonization temperature, the maximum adsorption capacity increased rapidly in the following order: MS700 (49016 mg·kg^−1^) > MS500 (28391 mg·kg^−1^) > MS350 (22987 mg·kg^−1^), and was consistent with the ref [39]. The adsorption capacities of the selected biochar samples were slightly higher than those of the biochar samples in Table 4, which were much higher than those of the four degraded soils; for example, the *Q*_m_ of MS700 was 288 times that of SDS. Hence, it is feasible to use stalk-derived biochar to increase the adsorption capacity and reduce the environmental mobility of Cd in degraded soil–biochar system.

### 3.4. The Adsorption Behavior of Cd on the Degraded Soils Amended by Biochars

**Figure 7:**
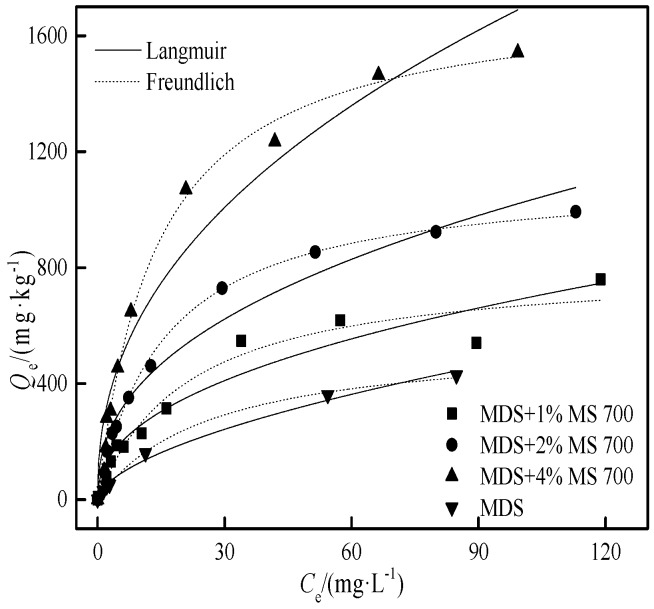
The effect of the weight ratio of MS700 on the Cd adsorption in a mixed system.

**Figure 8:**
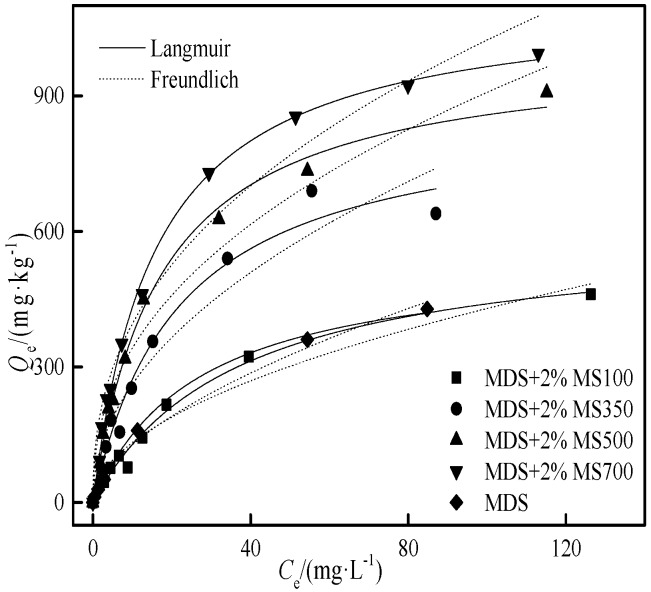
The effect of the biochar pyrolysis temperature on the Cd adsorption.

**Figure 9:**
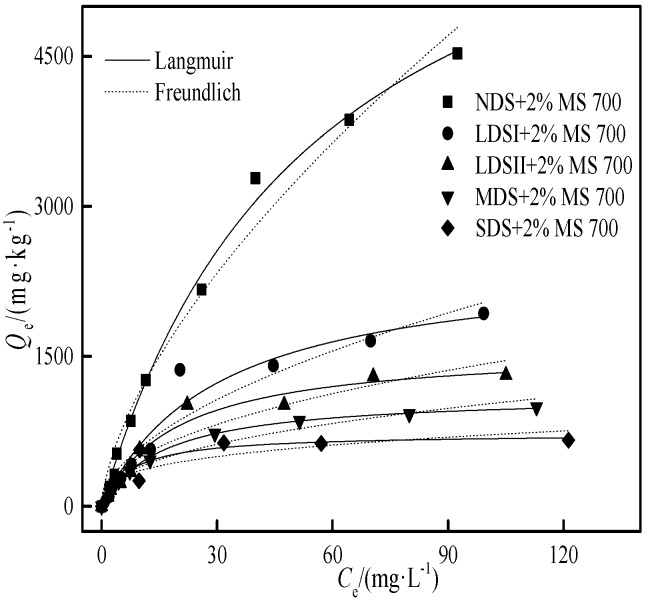
The Cd adsorption of the maize stalk biochar MS 700 in different type of soils.

Due to the cost and safety of biochar [42], it is usually applied in a relatively small quantity (≤5%) in soil amendment efforts. To make a more realistic assessment of the Cd adsorption in such a system, the adsorption behavior of Cd was studied in the following three systems: (1) The weight ratios of 1.0%, 2.0%, and 4.0% (wt) of MS700 were homogeneously mixed with moderately degraded soil; (2) 2.0% (wt) of the MS100 and biochars (MS350, MS500, MS700) in moderately degraded soil (MDS); (3) 2.0% (wt) MS700 in the soils with different degrees of degradation (LDSI, LDSII, MDS, SDS). The adsorption isotherm is shown in Figure 7, Figure 8 and Figure 9, and the regression parameters of the Langmuir and Freundlich adsorption models are shown in Table 5, Table 6 and Table 7.

#### 3.4.1. The Effect of Biochar Ratio on Cd Adsorption in Moderately Degraded Soil

With the increase of the biochar addition ratio (1%, 2%, and 4%), the *Q*_m_ values were 1.5, 2.0, and 3.2 times of pure MDS, whereas *K*_L_ was 1.3, 1.7, and 2.0 times that in MDS alone, respectively. It shows that the adsorption of Cd was significantly improved by the addition of biochar. The main adsorption contribution in amended degraded soil comes from adding biochar, and the *RC*_biochar_ (60.6–113%) increased with the increase of the biochar addition ratio. As the mass of biochar increased, the Cd adsorption of the amended MDS augmented, being comparable to the pure LDSI (1710 mg·kg^−1^).

Comparing the Cd adsorption data with those in the soil and biochar-alone systems in Table 3, the *Q*’_m,soil+MS_ calculated based on Equation (4) is much higher than the adsorption amount obtained in the experiment with the mixed soil and biochar system. In other words, the adsorption capacities of adsorbents were suppressed after the biochar was mixed with the soil. According to Equation (5), when the biochar presence in the soil was 1.0%, 2.0%, and 4.0%, the degree of decline on Cd adsorption (*D*) was 22.1%, 26.5%, and 30.1%, respectively. This indicates that the inhibition effect was enlarged when increasing the biochar ratio in the soil–biochar system. This is attributed to the physicochemical interactions between certain components of the soil and the adsorption sites of the biochar, that is, the pore blockage and surface coverage by the coated fine soil particles to the biochar. With the increase of the addition ratio, biochar particles were easy to be congregated and wrapped by the tiny particles of MDS which would heavily inhibit the adsorption capacity of the biochar.

#### 3.4.2. The Effect of Biochar Pyrolysis Temperature on Cd Adsorption in Moderately Degraded Soil

When adding 2% biomass (MS100) to moderately degraded soil, the adsorption capacity of the mixed system did not increase significantly (Table 6). When adding 2% biochars prepared at different pyrolysis temperatures (MS350, MS500, MS700), the adsorption capacity of the amended MDS increased significantly. With the increase of pyrolysis temperature of the biochar, the adsorption capacity of the degraded soil–biochar system was increased by 57%–100%, which is similar to the adsorption of Cd in the only-biochar system. The relative adsorption contribution of biochar and moderately degraded soil was similar in the mixed system while the relative adsorption contribution of biochar slightly increased with the increase of pyrolysis temperatures (Table 6).

The Langmuir model fits the experimental isotherm data better than the Freundlich model, *R*^2^ ≥ 0.98. Similar to the Section 3.4.1, the adsorption capacity of the moderately degraded soil and biochar mixed system was lower than the theoretically calculated *Q*’_m,soil+BC_ through Equation (4), indicating that the adsorption performance of the added biochar and moderately degraded soil was also suppressed. The adsorption decline level *D* in MDS with an addition of 2.0% of MS350, MS500, and MS700 were 13.3%, 9.4%, and 26.5%, respectively.

It is interesting to notice that the regression maximum adsorption amount obtained by the experiment isotherm was a little higher than the theoretically calculated *Q*’_m,soil+MS_ of MDS+2%MS100 based on Equation (4) (Table 6). That means the adsorption capacity of biomass and moderately degraded soil was not suppressed but accelerated when they were integrated with each other, which was much different from the soil–biochar system.

#### 3.4.3. The Cd Adsorption on Different Degraded Soils with 2.0% (wt) MS700

The experimental results show that the enhancement on Cd adsorption relatively increased with the level of soil degradation, that is, the *Q*_m_ values of the mixed system were increased by 36% (NDS+2%MS700), 44% (LDSI+2%MS700), 54% (LDSII+2%MS700), 102% (MDS+2%MS700), and 328% (SDS+2%MS700), compared with these soils alone. As the degree of soil degradation increased, the relative adsorption contribution of biochar in the mixed system gradually played a leading role (39.7%→135%).

**Figure 10:**
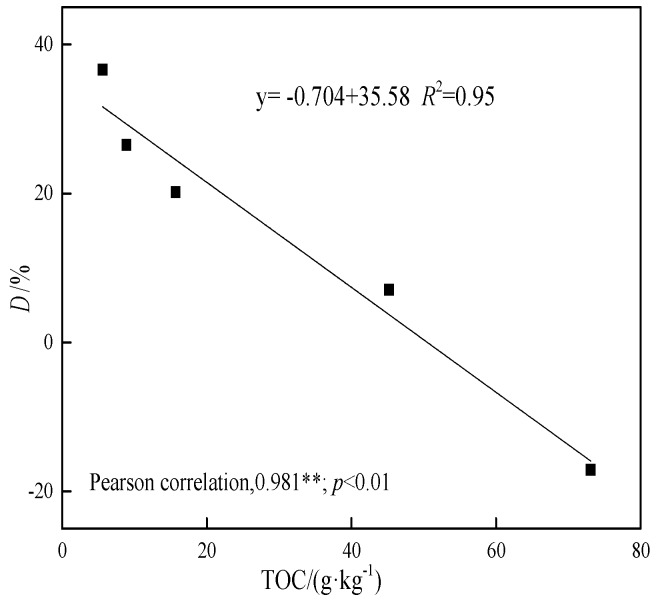
The relationship between the adsorption decline level (*D*) of Cd and the TOC of soil.

When the biochar was added to degraded soils, the Cd adsorption capacity of the mixed system was decreased to varying degrees. This is consistent with the observations in the previous two sets of adsorption experiments. The adsorption decline level *D* calculated by Equation (5) were 7.1%, 20.2%, 26.5%, and 36.6% in the degraded soils and biochar systems. It can be seen that the Cd adsorption of the mixed system was suppressed more when the soil degraded more seriously. The relationship between the adsorption decline level of Cd and the total organic carbon of soil is shown in Figure 10. The theoretically calculated *Q*’_m,soil+BC_ of NDS+2%MS700 is lower than the regression maximum adsorption amount obtained by the experiment isotherm (Table 7). That indicates the adsorption capacity of biochar and nondegraded soil was increased when they were incorporated with each other.

Summarily, the adsorption of Cd was slightly accelerated (7.1%) when biochar was incorporated with nondegraded soil but suppressed (7.1–36.6%) when biochar (different rate, different pyrolysis temperature) was added with degraded soils (different degradation degree). This phenomenon is different from the literatures (adsorption of heavy metal was usually increased in soil–biochar system) and it is worthy to discuss in detail the reasons and potential mechanisms of the inhibition and acceleration effect difference in the current system.

### 3.5. The Adsorption Inhibition and Acceleration Effect in Soil–Biochar System

**Figure 11:**
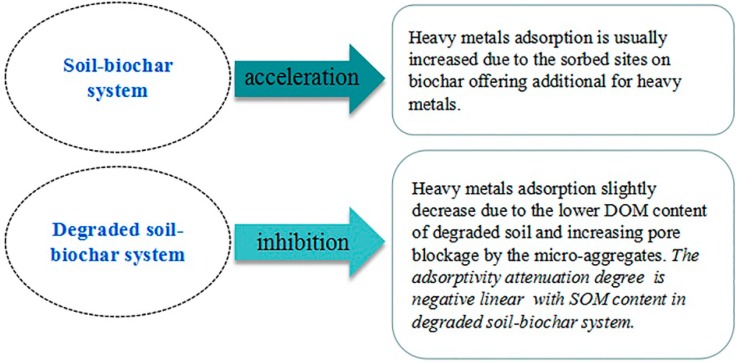
The adsorption inhibition and acceleration effect difference between soil–biochar system.

The inhibition and acceleration effect is one of the most critical factors when considering the biochar amendment to enhance the sorption and reduce the immobilization of soil contaminants. Generally, it is hard to calculate the sorption capacity of two sorbents though mechanical addition due to the inhibition or acceleration effect in bisorbents [43]. The sorption inhibition or acceleration effect of the soil–biochar system is influenced by the properties of sorbate pollutant (inorganic or organic, polar or nonpolar, aromatic or aliphatic), biochar (pyrolysis temperature and feedstocks), and soil (types, SOM content) [26]. The adsorption inhibition and acceleration effect difference between the soil–biochar system and the degraded soil–biochar system are summarized in Figure 11.

The slight enhancement of Cd adsorption was also reasonable when the high temperature biochar (MS700) was incorporated with nondegraded soil in the current study. DOM of nondegraded soil was expected to be effectively sorbed on MS700 due to its high surface area and low polarity. We were also excited to firstly find the inhibition effect occurred in adsorption of Cd in degraded soil–biochar. This seemingly abnormal phenomenon may be attributed to the limited DOM concentration of degraded soils and the pore-filling effect. The DOM concentration has been demonstrated to be significantly correlated with SOM content [44], and the DOM concentration is expected to decrease with soil degradation. The decline level of Cd adsorption in the degraded soil–biochar system is found to be negatively linear with the SOM content of soils (Figure 10). At the same time, the proportion of aggregates with small particles increases with soil degradation [42]. The tiny particles wrap around the surface of biochar and inactivate the adsorption sites of biochar, thus dropping the adsorption capacity of biochar [32].

## 4. Conclusions

With soil degradation, the Cd adsorption capacity of soil was significantly weakened (3–32 times). The addition of biochar enhanced the Cd adsorption capacity of the degraded soils (36–328%). The improving effect is stronger when increasing either the degradation level or the amount of added biochar, or the pyrolysis temperature of biochar. The main adsorption contribution mostly comes from biochar in the mixed system.

The adsorption of Cd was accelerated a bit when biomass was integrated with degraded soil or biochar was incorporated with nondegraded soil. Contrary to the general soil–biochar system, adsorption of Cd was not enhanced but slightly suppressed (7.1–36.6%) when biochar was incorporated with degraded soils. The adsorptivity attenuation degree is found to be negatively linear with SOM content in the degraded soil–biochar system, that is, the inhibition effect is stronger when the soil degrades more seriously.

## Figures and Tables

**Table 1:** The basic physicochemical properties of the six tested pristine soils.

Soil	Geographic Location	Moisture Content/%	pH	SSA/(m^2^·g^−1^)	TOC/(g·kg^−1^)
Peat ^1)^	N 33°10′23.0″, E 102°37′2.4″	88.26	5.72	3.10	92.7
NDS ^1)^	N 33°10′47.555″, E 102°37′34.172″	48.14	5.73	2.02	73.1
LDSI	N 33°19′36.0″, E 102°33′58.9″	33.47	6.03	2.52	45.2
LDSII ^1)^	N 33°10′45.710″, E 102°37′34.016″	10.53	6.40	2.71	15.7
MDS ^1)^	N 33°10′43.667″, E 102°37′33.488″	8.77	6.59	3.06	8.90
SDS	N 33°19′41.5″, E 102°33′42.5″	4.47	6.91	3.11	5.60

Notes: ^1)^ The data from the reference [32].

**Table 2:** The main physicochemical properties of the biochar.

Biochar	Yield/wt%	Ash/%	d_50_^1)^/μm	SSA/(m^2^·g^−1^)	Elemental Composition and Atomic Ratio of Biochar Organic Components ^2)^						pH
					C/wt%	H/wt%	N/wt%	O/wt%	(O+N)/C	H/C	
MS100	100.00	14.61	11.36	3.09	44.75	6.32	1.36	47.57	0.82	1.70	6.32
MS350	47.58	34.90	9.31	3.82	63.97	4.66	2.56	28.81	0.37	0.87	6.49
MS500	33.70	36.20	9.09	6.03	66.38	2.93	2.40	28.29	0.35	0.53	8.50
MS700	30.40	41.67	8.04	133.4	72.49	2.16	1.83	23.51	0.26	0.36	9.08

Notes: ^1)^ The d_50_ particle size is the equivalent diameter of the largest particle when the cumulative distribution in the distribution curve is 50%; ^2)^ The elemental composition of the organic component was obtained by subtracting the ash content and finally calculated as a mass fraction, where the percentage of O was calculated by subtraction.

**Table 3:** The parameters based on Langmuir and Freundlich model fittings for Cd adsorption isotherms with the six selected soils and the biochar.

Sample		Langmuir			Freundlich		
		*Q*_m_/(mg·kg^−1^)	*K*_L_/(L·mg^−1^)	*R* ^2^	*K*_f_/(mg·kg^−1^)(mg/L^−1^)^-N^	*N*	*R* ^2^
Soil ^1)^	Peat	6309	0.025	0.99	343	0.567	0.97
	NDS	5361	0.022	0.99	261	0.583	0.98
	LDSI	1710	0.054	0.99	126	0.555	0.98
	LDSII	1028	0.035	0.99	58	0.584	0.98
	MDS	553	0.037	0.99	33	0.583	0.99
	SDS	170	0.047	0.99	15	0.500	0.99
Biochar ^2)^	MS 100	2622	0.024	0.92	132	0.586	0.86
	MS 350	22987	0.030	0.99	1483	0.549	0.99
	MS 500	28391	0.061	0.97	3637	0.425	0.91
	MS 700	49016	0.047	0.98	4813	0.470	0.96

Notes: ^1)^ NDS: nondegraded soil; LDSI: slightly degraded soil I; LDSII: slightly degraded soil II; MDS: moderately degraded soil; SDS: severely degraded soil; ^2)^ Biochar derived from maize stalk with different pyrolysis temperature. The biomass samples were named as MS100, MS350, MS500, and MS700, respectively.

**Table 4:** Comparison adsorption capacity of maize stalk biochar for Cd with different adsorbents.

Adsorbent	Adsorption Capacity (*Q*_m_, mg·kg^−1^)	References
Vinegar residue biochar 700 °C	2910	[21]
Wheat straw biochar 450 °C	5000	[40]
Wheat straw biochar 600 °C	1960	[40]
Swine manure biochar 300 °C	42440	[40]
Peanut hull biochar 450 °C	6740	[41]
Maize stalk biochar 700 °C	49016	This study

**Table 5:** Regression parameters of Langmuir and Freundlich model fittings for Cd adsorption isotherms and calculated values on the MDS amended with different weight ratio of MS700.

Sample	*RC*^1)^ (%)		*D*^2)^ (%)	*Q’*_m,soil+MS_^3)^/(mg·kg^−1^)	Langmuir			Freundlich		
	*RC*_soil_	*RC*_biochar_			*Q*_m_/(mg·kg^−1^)	*K*_L_/(L·mg^−1^)	*R* ^2^	*K*_f_/(mg·kg^−1^)(mg·L^−1^)^−N^	*N*	*R* ^2^
MDS	100	--	--	--	553	0.037	0.99	33.3	0.583	0.99
MDS+1%MS 700	67.7	60.6	22.1	1038	809	0.047	0.95	93.7	0.434	0.93
MDS+2%MS 700	48.5	87.7	26.5	1522	1118	0.063	0.99	153	0.413	0.97
MDS+4%MS 700	30.5	113	30.1	2492	1742	0.072	0.99	231	0.432	0.95

Notes: ^1)^ The adsorption contribution rate *RC* calculated by Equation (6); ^2)^ The adsorption decline level *D* calculated by Equation (5); ^3)^ The *Q’*_m,soil+MS_ calculated by Equation (4).

**Table 6:** The regression parameters of Langmuir and Freundlich model fittings for Cd adsorption isotherms and calculated values on the soil amended with biochar prepared at different temperatures.

Sample	*RC*^1)^ (%)		*D*^2)^ (%)	*Q’*_m,soil+MS_^3)^/(mg·kg^−1^)	Langmuir			Freundlich		
	*RC*_soil_	*RC*_biochar_			*Q*_m_/(mg·kg^−1^)	*K*_L_/(L·mg^−1^)	*R* ^2^	*K*_f_/(mg·kg^−1^)(mg·L^−1^)^−N^	*N*	*R* ^2^
MDS	100	--	--	--	553	0.037	0.99	33.3	0.583	0.99
MDS+2%MS 100	90.2	8.73	−1.2	594	601	0.027	0.99	41.1	0.509	0.95
MDS+2%MS 350	62.4	52.9	13.3	1002	869	0.046	0.98	86.7	0.480	0.93
MDS+2%MS 500	53.9	56.4	9.4	1110	1006	0.058	0.99	129	0.424	0.97
MDS+2%MS 700	48.5	87.7	26.5	1522	1118	0.063	0.99	153	0.413	0.97

Notes: ^1)^ The adsorption contribution rate *RC* calculated by Equation (6); ^2)^ The adsorption decline level *D* calculated by Equation (5); ^3)^ The *Q’*_m,soil+MS_ calculated by Equation (4).

**Table 7:** The regression parameters of Langmuir and Freundlich model fittings for adsorption isotherms and calculated values for Cd on soils amended with 2.0% MS700.

Sample	*RC*^1)^ (%)		*D*^2)^ (%)	*Q’*_m,soil+MS_^3)^/(mg·kg^−1^)	Langmuir			Freundlich		
	*RC*_soil_	*RC*_biochar_			*Q*_m_/(mg·kg^−1^)	*K*_L_/(L·mg^−1^)	*R* ^2^	*K*_f_/(mg·kg^−1^)(mg·L^−1^)^-N^	*N*	*R* ^2^
NDS+2%MS 700	72	13.4	−17.1	6234	7300	0.018	0.99	265	0.639	0.98
LDSI+2%MS 700	67.9	39.7	7.1	2656	2468	0.033	0.96	170	0.540	0.92
LDSII+2%MS 700	63.5	61.8	20.2	1988	1586	0.051	0.97	171	0.461	0.92
MDS+2%MS 700	48.5	87.7	26.5	1522	1118	0.063	0.99	153	0.413	0.97
SDS+2%MS 700	22.9	134.8	36.6	1147	727	0.134	0.89	180	0.299	0.8

Notes: ^1)^ The adsorption contribution rate *RC* calculated by Equation (6); ^2)^ The adsorption decline level *D* calculated by Equation (5); ^3)^ The *Q’*_m,soil+MS_ calculated by Equation (4).
